# Corneal and Endothelial Parameters Following Scleral Lens Wear in Post-Keratoplasty Eyes

**DOI:** 10.1007/s44402-026-00111-6

**Published:** 2026-06-11

**Authors:** Ankit Raj, Ankita Kumari, Srikanth Dumpati, Mark Willcox, Stephen J. Vincent, Mukesh Kumar

**Affiliations:** 1 LV Prasad Eye Institute, Hyderabad, India; 2 School of Optometry and Vision Science, University of New South Wales, Sydney, New South Wales Australia; 3 Contact Lens and Visual Optics Laboratory, Optometry and Vision Science, Centre for Vision and Eye Research, Queensland University of Technology, Brisbane, Queensland Australia

**Keywords:** Corneal oedema, Endothelium, Scleral contact lens, Specular microscope

## Abstract

**Purpose:**

To investigate the changes in corneal endothelial morphology and central corneal thickness following short-term scleral lens wear in eyes that had undergone penetrating keratoplasty (PK) and deep anterior lamellar keratoplasty (DALK), and to examine the association between baseline endothelial characteristics and the magnitude of scleral lens–induced corneal oedema.

**Methods:**

Twelve eyes were fitted with non-fenestrated scleral lenses (oxygen permeability [Dk] 100; centre thickness ≈ 250 µm). Endothelial cell density (ECD), coefficient of variation in cell size (CoV), percentage of hexagonal cells (% Hex) and central corneal thickness (CCT) were measured before and immediately after 8 h of scleral lens wear using specular microscopy.

**Results:**

Following lens wear, ECD (2041 ± 557 vs 1827 ± 481 cells/mm²) and % Hex (44.4 ± 12.0% vs 38.2 ± 10.0%) decreased, while CoV (37.3 ± 10.3% vs 42.4 ± 9.4%) and CCT (485.3 ± 47.1 vs 520.4 ± 51.3 µm, 7.2 ± 3.1% oedema) increased (all *p* ≤ 0.001). These short‑term specular microscopy-derived changes in ECD and endothelial morphology may be an imaging artefact associated with transient corneal swelling rather than true endothelial cell loss. The magnitude of corneal oedema was not associated with any baseline corneal endothelial parameters (*p* > 0.20) but correlated with graft age (*r* = 0.80). Corneal oedema was greater in PK than DALK eyes (11.38% vs 5.23%, *p* = 0.003), and PK grafts were older on average (7 vs 5 years).

**Conclusions:**

Short-term scleral lens wear induced significant corneal swelling in post-keratoplasty eyes but was not associated with baseline endothelial parameters.

Key Points

- Scleral lens wear induces significant central corneal oedema in post-keratoplasty eyes.
- Rapid changes in endothelial parameters are likely artefacts from transient corneal swelling, not true cell loss.
- Oedema is unrelated to baseline endothelial parameters but increases with graft age and is greater in penetrating keratoplasty than deep anterior lamellar keratoplasty eyes.

## Introduction

The corneal endothelium is essential for maintaining stromal hydration and corneal transparency [1]. Endothelial morphology is commonly evaluated using three key indicators: endothelial cell density (ECD), which represents the number of cells per square millimetre; the coefficient of variation (CoV) in cell area, which reflects variation in cell size and the percentage of hexagonal cells (% Hex), which indicates the regularity of cell shape [2]. When endothelial cells are lost, neighbouring cells enlarge and redistribute to preserve the integrity of the monolayer [1]. This compensatory response is often uneven, resulting in increased variability in cell size (polymegathism) and a reduction in hexagonal cell proportion (pleomorphism) [3]. Previous investigations have demonstrated that subtle alterations in corneal endothelial cell morphology serve as an early indicator of endothelial stress and structural instability [4, 5]. For example, contact lens wear causes a decrease in ECD and an increase in polymegathism and pleomorphism [6, 7].

Visual rehabilitation following penetrating keratoplasty accounts for ~17% of scleral lens fits [8]. These lenses offer significant optical benefits in post-graft eyes by neutralising the high levels of irregular corneal astigmatism commonly encountered after surgery and reducing anisometropia and aniseikonia in unilateral cases [9–11]. In addition, since scleral lenses vault the cornea and land on the conjunctiva, they provide superior on-eye stability compared with corneal rigid lenses and allow complete clearance of the graft–host interface. This minimises mechanical interaction with the cornea, thereby reducing the likelihood of irritation and inflammatory responses during wear.

Despite these fitting and optical advantages, the risk of scleral lens-induced corneal oedema remains a major clinical concern in post-graft eyes due to the progressive decline in ECD that typically follows penetrating keratoplasty [12]. For example, endothelial cell loss may reach 10 to 30% within the first postoperative year and can exceed 50% over a 5-year period, substantially reducing the cornea’s capacity to regulate hydration [13]. Corneal complications can arise in post-graft eyes fitted with scleral lenses, such as stromal and epithelial oedema due to hypoxia or mechanical touch [14, 15].

In a study of short-term scleral lens wear in post-penetrating keratoplasty eyes, Schear et al. reported a 15% failure rate, predominantly in cases with severely reduced (and often unmeasurable) endothelial cell counts [16]. In unsuccessful fits, corneal oedema reached approximately 18% after only 2 h of wear, despite the use of highly oxygen-permeable lens materials. While a number of scleral lens fitting characteristics have been shown to influence the magnitude of corneal oedema in healthy eyes such as the lens material [17], as well as both the lens [18] and fluid reservoir thickness [19], in the post graft eye, corneal endothelial function is likely a critical factor, as noted in previous research of healthy adults following overnight scleral lens wear [20].

To date, only two studies have quantified scleral lens–induced corneal swelling in eyes following penetrating keratoplasty. One retrospective analysis reported approximately 4% oedema after 6 h of wear, based on ultrasound-derived measurements [21]. The second investigation demonstrated significant central and mid-peripheral corneal oedema, with greater thickening toward the graft–host junction and increased variability compared with healthy eyes [22]. However, neither study evaluated corneal endothelial metrics such as ECD, polymegathism, or pleomorphism.

Therefore, the aim of this study was to evaluate the change in corneal endothelial morphology following short-term scleral lens wear in eyes that had undergone deep anterior lamellar keratoplasty (DALK) and penetrating keratoplasty (PK), and to examine the association between endothelial morphology and the extent of scleral lens-induced corneal oedema.

## Methods

This prospective, observational study was approved by the Human Ethics Research Committee of the University of Health Sciences, India, conducted at a tertiary eye hospital affiliated with the university and adhered to the tenets of the Declaration of Helsinki. Following an explanation regarding the nature of the experiment, all participants provided informed consent.

### Participants

Twelve adults (mean age (standard deviation) 28.7 (4.8) years; eight females and four males) with a history of keratoconus who had previously undergone corneal transplantation (eight deep anterior lamellar keratoplasty and four full-thickness penetrating keratoplasty) were recruited for scleral lens fitting (Table 1). All participants were non–contact lens wearers at the initial screening. Although rigid gas permeable lenses had previously been trialled, they were not tolerated, and scleral lenses were considered the most suitable option. An ophthalmic screening examination was conducted to exclude participants with active anterior segment disease, ocular allergy, eyelid disease or any contraindication to contact lens wear. Visual acuity was measured using an Early Treatment of Diabetic Retinopathy Screening chart under standardised illumination. The study was conducted over two visits separated by 1 week.

**Table 1:** Overview of the study participants including demographics, surgical details, scleral lens parameters, wearing time and central corneal oedema %.

Participants	Sex	Eye	Age	Graft type	Time since surgery(y)	Back vertex power(D)	Sagittal depth (mm)	Initial fluid reservoir thickness (µm)	Wearing time (hr)	Central corneal oedema (%)
**1**	M	R	28	DALK	5	−5.00	5.41	250	8.50	5.10
**2**	M	R	25	DALK	4	−5.00	4.91	251	7	2.29
**3**	F	L	31	PK	6	−6.00	5.59	255	9	8.05
**4**	M	R	25	DALK	3	−8.00	5.68	249	8.50	6.32
**5**	F	L	33	DALK	5	−5.00	5.41	255	8	6.07
**6**	F	R	28	PK	7	−8.00	5.68	258	7.00	9.44
**7**	F	L	31	DALK	6	−4.00	5.19	251	8.50	5.70
**8**	M	R	26	DALK	5	−4.00	5.19	256	9	6.37
**9**	F	R	27	DALK	5	−5.00	5.41	248	8	5.10
**10**	F	R	24	DALK	4	−6.00	5.59	251	8.50	4.87
**11**	F	R	25	PK	6	−12.00	6.30	247	7.75	10.32
**12**	F	R	41	PK	8	−12.00	6.30	250	9	17.71

*DALK* deep anterior lamellar keratoplasty, *F* female, *L* left eye, *M* male, *PK* penetrating keratoplasty, *R* right eye.

### Scleral Lens Design and Fitting

A comprehensive anterior segment assessment was performed to confirm eligibility and ensure there were no contraindications to scleral lens wear. Keracare (Acculens, acculens.com) scleral diagnostic lenses (non-fenestrated, roflufocon D; Dk 100; approximately 250 µm centre thickness) with a standard spherical landing zone were used to determine an appropriate fitting lens. A trial set of 10 lenses with overall diameters of 15.9 mm and 16.4 mm was used, with all lenses incorporating a standard spherical landing zone design. The overall diameter was selected to ensure that the lens extended at least 2 mm beyond the limbus. All fittings were conducted by a single experienced optometrist following the manufacturer’s guidelines.

Lenses were applied using sterile saline (LacriPure®, Menicon Co.,, menicon.com) and sodium fluorescein (Fluorets, ophthalmic strips 1 mg, Contacare Ophthalmic, optitecheyecare.com). The target post-lens fluid reservoir thickness was approximately 250 µm, and was measured immediately after lens insertion using anterior segment optical coherence tomography (OCT) (Optovue Inc. visionix.com).

Landing zone alignment was evaluated using slit-lamp biomicroscopy (SL-1E, Topcon, topconhealthcare.com) to confirm the absence of conjunctival blanching and vascular compression. Following confirmation of satisfactory central clearance and haptic alignment, lenses were allowed to settle for 1 h, after which the centration, fluid reservoir thickness and the presence of air bubbles were reassessed using a slit-lamp.

### Experimental Details

A second study visit was conducted 1 week later using the optimum fitting diagnostic scleral lens determined at the previous fitting visit; no contact lenses were worn by participants between the study visits to allow resolution of any transient corneal effects before the experimental wear session [23]. ECD, CoV, percentage of hexagonal cells (%) and central corneal thickness (CCT) was evaluated by a masked examiner using a non-contact specular microscope (SP-3000P; Topcon, topconhealthcare.eu) before and immediately after a period of scleral lens wear of at least 7 h (mean wearing time, 8.2 h [95% confidence interval, 7.7 to 8.7 h]; range, 7 to 9 h). Images with inadequate clarity or poorly delineated endothelial cell borders were excluded and reacquired. For each eye, three consecutive measurements were obtained, and the mean value was used for analysis. Participants attended a single session on the second visit, during which both baseline measurements (prior to scleral lens application) and post-wear measurements were performed on the same day. Baseline measurements were obtained in the morning (08:00–10:00 h) prior to scleral lens application and after 8 h of lens wear (16:00–18:00 h), at which time repeat measurements were performed immediately following scleral lens removal.

Endothelial analysis was conducted using the IMAGEner 2000 software, topconhealthcare.eu, which automatically identifies the analysis area and provides high-resolution evaluation of the endothelial mosaic. The image analysis was performed by a single examiner who was masked to the pre- and post-lens wear measurements. After the manual selection of 50 contiguous cells, the software calculated the ECD, CoV, % and CCT (µm). The change in CCT is presented as the percentage change relative to the pre-lens wear measurement.

### Statistical Analyses

The required sample size was estimated using previously published data on the short-term corneal response to scleral lens wear in post-keratoplasty eyes, which reported a mean increase in corneal thickness of 2.99% [22]. Using these parameters, with a type I error of 0.05 and statistical power of 80%, a minimum of 10 participants was required to detect pre- to post-lens differences in CCT. Statistical analyses were conducted using IBM SPSS software (version 26.0; ibm.com) and GraphPad Prism (version 10.0; GraphPad Software, graphpad.com). Data normality was evaluated using the Shapiro–Wilk test. Depending on the distribution of the data, pre- and post-lens wear comparisons were performed using either paired *t*-tests or Wilcoxon signed-rank tests. Pearson correlation analyses were used to examine associations between changes in endothelial parameters and other clinical variables. A two-tailed significance threshold of *p* < 0.05 was considered statistically significant for all analyses.

## Results

Significant changes were observed in all corneal endothelial parameters following 8 h of scleral lens wear, including a decrease in ECD and hexagonality, with an increase in cell size variability and CCT (all *p* < 0.001; Table 2).

**Table 2:** Comparison of corneal endothelial morphologic parameters and central corneal thickness and immediately after removal of scleral lenses following 8 h of wear.

	Endothelial cell density (cell/mm^2^)		Coefficient of variation		Hexagonality (%)		Central corneal thickness (µm)	
	Before	After	Before	After	Before	After	Before	After scleral lens
Mean	2041 ± 557	1827 ± 481	37 ± 10	42 ± 9	44 ± 12	38 ± 10	485 ± 47	520 ± 51
Median	2199	1996	36	43	42	37	485	520
Range	1166 to 2601	1001 to 2328	24 to 55	30 to 58	24 to 65	23 to 56	392 to 567	412 to 580
*P* value^a^	0.001		0.001		0.001		0.0001	

^a^Wilcoxon Signed-rank test.

**Fig. 1:**
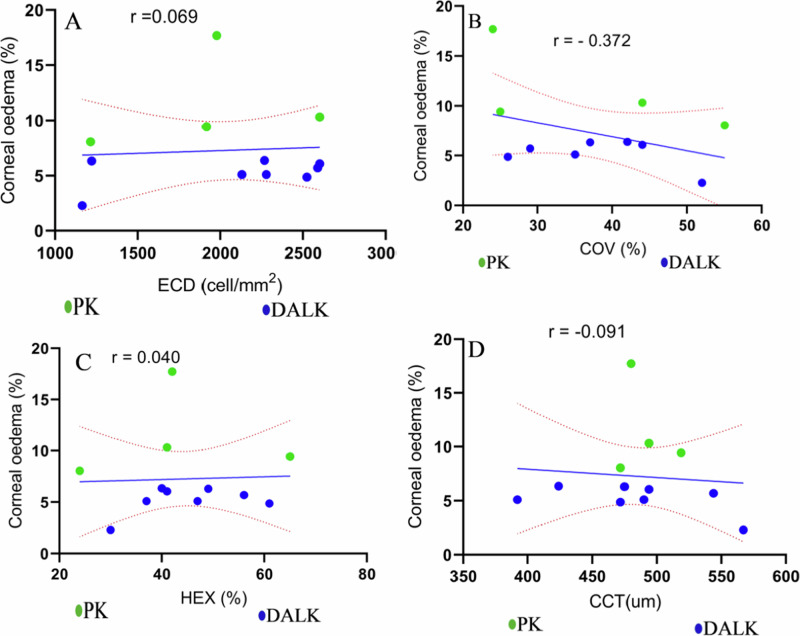
Associations between corneal oedema, expressed as the percentage change in central corneal thickness and baseline corneal endothelial morphology parameters measured before scleral lens wear. Scatter plots illustrate relationships between corneal oedema and **A** ECD (endothelial cell density; cells per square millimetre), **B** COV (coefficient of variation in endothelial cell size), **C** HEX (percentage of hexagonal endothelial cells) and **D** CCT (central corneal thickness; micrometres). DALK deep anterior lamellar keratoplasty, PK penetrating keratoplasty. The blue line represents the linear regression line, and the red line is the 95% confidence interval.

Correlation analyses revealed no significant associations between the magnitude of corneal oedema and baseline ECD, CoV in cell size, % or CCT, either in the overall grafted cohort or when analysed separately for the penetrating keratoplasty (PK *n* = 4) and deep anterior lamellar keratoplasty (DALK *n* = 8) eyes (all *p* > 0.20; Table 3, Fig. 1). These findings indicate that baseline endothelial morphology was not significantly associated with the magnitude of corneal oedema following short-term scleral lens wear.

**Table 3:** Correlation between corneal oedema (post–pre CCT, %) and baseline corneal endothelial parameters (pre-scleral lens wear) in total grafted eyes, penetrating keratoplasty (PK) and deep anterior lamellar keratoplasty (DALK).

Group		ECD (cells/mm^2^)	COV%	HEX%	CCT (µm)
All eyes	*r*	0.069	−0.372	0.040	0.091
	95% CI	−0.525 to 0.618	−0.779 to 0.255	−0.545 to 0.600	−0.632 to 0.508
	*R*^2^	0.004	0.139	0.001	0.90
	*p*-value	0.83	0.23	0.90	0.77
PK	*r*	0.271	−0.644	0.075	−0.238
	95% CI	−0.933 to 0.977	−0.991 to 0.831	−0.954 to 0.966	−0.975 to 0.937
	*R*^2^	0.073	0.415	0.005	0.057
	*p*-value	0.73	0.36	0.93	0.76
DALK	*r*	0.455	−0.410	0.420	−0.510
	95% CI	−0.367 to 0.878	−0.865 to 0.413	−0.404 to 0.867	−0.893 to 0.302
	*R*^2^	0.207	0.168	0.176	0.260
	*p*-value	0.26	0.31	0.30	0.20

Corneal oedema was expressed as the percentage change in CCT following scleral lens wear. Pearson correlation analysis was performed (*n* = 12).

*CCT* central corneal thickness, *CI* confidence interval, *CoV* coefficient of variation in cell size, *ECD* endothelial cell density, *HEX* (percentage of hexagonal endothelial cells).

A strong positive correlation was observed between graft age and the magnitude of corneal oedema (*r* = 0.80). However, this association may be partially confounded by the type of keratoplasty performed, as PK grafts were older on average than DALK grafts (mean graft age: 7 vs 5 years, respectively). Consistent with this, the magnitude of corneal oedema was greater in PK eyes compared with DALK eyes (mean ± SEM: 11.38% vs 5.23%; unpaired *t*-test, *t* (10) = 3.85, *p* = 0.003). These findings suggest that both graft age and surgical procedure may contribute to the observed oedematous response.

## Discussion

This study identified statistically significant changes in all corneal endothelial parameters assessed following short-term scleral lens wear in post-keratoplasty eyes. Similar reductions in ECD have previously been reported with long-term soft contact lens wear [24]. Although the magnitude of the change observed in the present study was modest, it may reflect transient alterations in endothelial cell configuration related to short-term physiological stress, or an imaging artefact, rather than permanent endothelial cell loss [25]. A previous study reported endothelial bleb formation following scleral lens wear with thicker fluid reservoirs (400 µm), indicating greater endothelial stress due to reduced oxygen availability compared with lower clearances (200 µm). In the present study, the post-lens fluid reservoir was approximately 250 µm, representing a moderate clearance that is closer to the lower-clearance range. This fitting strategy may have mitigated hypoxia-related endothelial stress while maintaining adequate corneal clearance, thereby supporting corneal physiological stability [26]. In parallel, the CoV in cell size increased, suggesting greater heterogeneity in endothelial cell area after lens wear. A concomitant reduction in hexagonality was also noted, indicating a disruption in the regular endothelial cell mosaic. The structural uniformity of corneal endothelial cells plays a critical role in preserving corneal transparency and stromal hydration, and deviations in cell morphology may progressively impair these functions [27]. These findings are consistent with reports from studies investigating long-term soft contact lens wear, where increased polymegathism and reduced hexagonality have been observed [24]. However, it should be noted that corneal swelling can impact endothelial measurements obtained using specular microscopy (e.g., ECD) [16, 28], and the findings should be interpreted with caution.

An increase in CCT was observed following scleral lens wear, consistent with previous reports. A single case report has similarly described corneal oedema in a post-PK cornea associated with scleral lens wear, suggesting that altered corneal hydration during lens wear may be related to reduced oxygen availability or limited tear exchange beneath the scleral lens [29]. However, given the short duration of wear in the current study, these changes are likely to be reversible and should be interpreted with caution. A previous study showed that grafted corneas deswelled significantly more slowly than fellow unoperated corneas following hypoxic oedema, suggesting alterations in endothelial barrier function and/or reduced pump efficiency [30]. An earlier investigation demonstrated a modest but measurable increase in CCT following approximately 2 h of scleral lens wear, consistent with mild lens-induced corneal oedema. The magnitude of swelling was greater in post-graft corneas (3.51%) than in irregular (0.84%) and regular corneas (1.7%), suggesting that grafted corneas may exhibit increased vulnerability to hypoxia-related stromal changes during scleral lens wear [31]. A previous retrospective study [21] reported central corneal oedema following scleral lens wear in post–PK eyes, with an average increase of approximately 1.8% after 6 h of wear. In the present study, a greater magnitude of scleral lens–induced central corneal oedema was observed, particularly in penetrating keratoplasty eyes (11.4%) compared with DALK eyes (5.3%). This discrepancy may be influenced by differences in endothelial involvement, lens characteristics, wear duration and instrumentation used to measure corneal thickness [32].

In addition, the association between baseline endothelial morphology and the magnitude of scleral lens-induced corneal oedema was examined. Specifically, correlations were assessed between pre-lens wear ECD, CoV, % HEX and CCT with the percentage of corneal oedema induced by scleral lens wear. No statistically significant correlations were identified for any of these parameters. In post–PK eyes wearing scleral lenses, lower oxygen transmissibility (Dk/t) of the scleral–tear lens system has been associated with greater corneal swelling after 6–8 h of wear, with significantly higher swelling observed in eyes fitted with Dk/*t* < 20 compared with those with Dk/*t* > 20, despite generally low central corneal clearance. Interestingly, ECD was not significantly associated with the magnitude of corneal swelling within the clearance range studied, underscoring the role of lens-related factors in short-term scleral lens-induced corneal oedema in post-graft eyes [33]. These findings suggest that, within this cohort, baseline endothelial morphological characteristics and CCT were not predictive of the extent of short-term corneal oedema induced by scleral lens wear. This lack of association may indicate that scleral lens-related corneal swelling is influenced more by lens-related factors (such as lens-fit, reservoir characteristics or oxygen transmissibility) or transient physiological responses, rather than the pre-existing endothelial morphology. Similar observations have been reported in previous studies, where corneal swelling during scleral lens wear was primarily attributed to lens design and fitting characteristics rather than corneal endothelial parameters [22]. In the present study, scleral lens–induced corneal oedema was greater in PK eyes than in DALK eyes, likely reflecting differences in surgical technique and postoperative endothelial reserve. This is supported by the lower mean ECD observed in PK eyes (1928 ± 566 cells/mm²) compared with DALK eyes (2097 ± 582 cells/mm²). Previous studies have demonstrated substantial endothelial cell loss following PK, with Obata et al. [34] reporting losses approaching 48% at 12 months. Longitudinal keratoconus cohorts demonstrate divergent postoperative outcomes between PK and DALK, with PK grafts showing progressive corneal thickening over time, whereas DALK grafts typically stabilise after the early postoperative period, consistent with reduced chronic stromal oedema when the host endothelium is preserved [35, 36]. Contralateral-eye and same-size graft studies further report greater long-term endothelial cell loss in PK compared with DALK, placing PK grafts closer to endothelial decompensation at comparable graft ages [36, 37]. These findings are consistent with the present study, in which PK eyes demonstrated greater corneal oedema than DALK eyes. However, to date, no studies have specifically evaluated changes in corneal endothelial morphology following scleral lens wear in both PK and DALK eyes.

While the present study evaluated short-term responses to scleral lens wear following penetrating keratoplasty, longer-term outcomes have been described in retrospective studies. Severinsky et al. [38] reported sustained scleral lens wear over a mean follow-up of 5 years in most post-keratoplasty eyes, despite intermittent episodes of graft rejection and corneal oedema. Lens discontinuation due to corneal decompensation was uncommon, particularly when scheduled lens removal was employed to enhance corneal oxygenation. Other reports described scleral lens dropout rates of up to 35%, often related to handling difficulties rather than corneal complications alone [39–41]. The present study demonstrates short-term changes in ECD, CoV, % and CCT following scleral lens wear in post-keratoplasty eyes, including both PK and DALK. However, the lack of association between baseline endothelial parameters and the magnitude of corneal oedema suggests that endothelial morphology alone has limited utility in predicting individual corneal responses to scleral lens wear. These findings indicate that a short-term scleral lens wear assessment may be a clinically relevant approach to evaluating oedema and guiding scleral lens fitting in grafted corneas.

Although scleral lens-associated corneal oedema in post- PK eyes can be clinically significant, lens prescribing should be guided by a thorough assessment of the risk–benefit profile, weighing possible long-term corneal compromise against the potential gains in vision and quality of life. To mitigate hypoxic and mechanical stress during scleral lens wear in post-PK corneas, several fitting strategies are recommended. These include utilising lens materials with high oxygen permeability, optimising central lens thickness [42, 43] to the minimal level compatible with optical and structural requirements and reducing the post-lens tear reservoir thickness [19]. Additional measures, such as restricting duration or implementing regular intervals, may further limit corneal oedema. The use of fenestrated or channelled scleral lens designs may also be advantageous, as their reduced central clearance, enhanced tear exchange and diminished suction effect may collectively reduce corneal hypoxic stress in post-graft eyes [44].

The major limitations of this study include the relatively small sample size, which may limit the generalisability of the findings to the broader post–penetrating keratoplasty population. In addition, a uniform spherical landing zone design was used for all participants, which can result in reduced tear exchange and potentially greater hypoxia [45]. Consequently, the extent of corneal oedema reported in this study may represent an overestimation compared with that which could occur with other landing zone designs. Second, measurements were limited to an 8-hour wearing period; therefore, the findings may not be generalisable to habitual or long-term scleral lens wear. A further limitation is that central scleral lens thickness was not quantified with OCT, preventing precise estimation of oxygen transmissibility at the corneal surface, although all lenses were manufactured in roflufocon D (Dk 100) with a standardised design and power profile, making clinically meaningful variability in oxygen transmissibility between lenses unlikely. In addition, the inability to measure from exactly the same corneal location during both measurements and the absence of post-lens removal follow-up preclude confirmation of whether the observed corneal changes were transient or fully reversible.

Future studies should include larger cohorts and longitudinal designs to assess the persistence, reversibility or adaptation of endothelial and corneal thickness changes with repeated or long-term scleral lens wear. Incorporating serial post-wear measurements would help clarify recovery kinetics. Further work should also examine the influence of lens design parameters, tear reservoir thickness and oxygen transmissibility on corneal physiological responses.

## Conclusion

Short-term scleral lens wear in eyes that had undergone DALKor PK was associated with alterations in specular microscopy–derived endothelial parameters. These changes should be interpreted with caution, as the study design did not permit differentiation between true endothelial morphological alterations and transient or measurement-related effects secondary to corneal hydration. Baseline endothelial parameters were not associated with the magnitude of post-wear corneal oedema. Careful pre-fitting assessment and ongoing monitoring of corneal thickness and endothelial characteristics are recommended when prescribing scleral lenses in grafted corneas.

## Data Availability

No datasets were generated or analysed during the current study.
